# Surface-Enhanced Raman Spectroscopy on Hybrid Graphene/Gold Substrates near the Percolation Threshold

**DOI:** 10.3390/nano10010164

**Published:** 2020-01-17

**Authors:** Dmitry E. Tatarkin, Dmitry I. Yakubovsky, Georgy A. Ermolaev, Yury V. Stebunov, Artem A. Voronov, Aleksey V. Arsenin, Valentyn S. Volkov, Sergey M. Novikov

**Affiliations:** 1 Center for Photonics and 2D Materials, Moscow Institute of Physics and Technology (MIPT), 141700 Dolgoprudny, Russia; dmitrii.yakubovskii@phystech.edu (D.I.Y.); or stebunov@phystech.edu (Y.V.S.); voronov.artem@gmail.com (A.A.V.); arsenin.av@mipt.ru (A.V.A.); vsv.mipt@gmail.com (V.S.V.); 2 Skolkovo Institute of Science and Technology, 121205 Moscow, Russia

**Keywords:** surface-enhanced Raman scattering, graphene, ultrathin gold films, spectroscopic ellipsometry, percolation threshold

## Abstract

Graphene is a promising platform for surface-enhanced Raman spectroscopy (SERS)-active substrates, primarily due to the possibility of quenching photoluminescence and fluorescence. Here we study ultrathin gold films near the percolation threshold fabricated by electron-beam deposition on monolayer CVD graphene. The advantages of such hybrid graphene/gold substrates for surface-enhanced Raman spectroscopy are discussed in comparison with conventional substrates without the graphene layer. The percolation threshold is determined by independent measurements of the sheet resistance and effective dielectric constant by spectroscopic ellipsometry. The surface morphology of the ultrathin gold films is analyzed by the use of scanning electron microscopy (SEM) and atomic force microscopy (AFM), and the thicknesses of the films in addition to the quartz-crystal mass-thickness sensor are also measured by AFM. We experimentally demonstrate that the maximum SERS signal is observed near and slightly below the percolation threshold. In this case, the region of maximum enhancement of the SERS signal can be determined using the figure of merit (FOM), which is the ratio of the real and imaginary parts of the effective dielectric permittivity of the films. SERS measurements on hybrid graphene/gold substrates with the dye Crystal Violet show an enhancement factor of ~10^5^ and also demonstrate the ability of graphene to quench photoluminescence by an average of ~60%.

## 1. Introduction

Surface-enhanced Raman spectroscopy (SERS) [1,2,3,4] is a powerful and highly selective tool that allows researchers to identify chemical compounds and determine the structure of materials and molecules based on their specific vibration bonds. SERS utilizes strong electromagnetic field enhancement (FE) which occurs due to resonantly excited surface plasmons, i.e., collective electron oscillations on a metal surface coupled to electromagnetic fields in dielectric media [5,6,7,8]. The spectral position of the resonances is tunable through a variety of parameters, such as metal, geometry, the composition of nanostructures [9,10], or also size and shape, in the case of nanoparticles (NPs) [6,7,11,12]. Plasmonic nanostructures can be used for SERS applications, thus allowing for the detecting of molecules at very low concentrations (in the order of nM) and is of great interest in practical developments in the field of sensory, biochemistry, and medical diagnostics [1,13,14,15,16].

Various strategies have been suggested to realize a strong and robust FE effect. One of the ways to get strong FE is to create structures with so-called “hot spots” [17,18,19] that are formed in the interparticle spaces of about 1–3 nm. One of the easiest and cheapest methods for the fabrication of large-area SERS substrates relies on the usage of semi-continuous metal films near the percolation threshold [20,21,22], the critical point at which individual metal clusters start forming connected structures across the substrate domains [20,23,24]. The concentration of such hot spots (along with the average value of the SERS signal gain) is expected to be maximal near the percolation threshold [22,25]. These films are usually prepared by the high-vacuum evaporation of a noble metal (gold, silver, or copper) onto a supporting substrate (semiconductor or dielectric). The presence of two-dimensional materials on the surface of the substrate leads to a change in the kinetics of metal growth, which affects the optical properties of the thin metal films [26,27] and the critical thickness that determines the percolation threshold. These changes provide additional options for modifying SERS substrates. In addition, the usage of two-dimensional materials such as graphene can significantly improve the performance of SERS substrates and extend their in vivo applications [28,29]. Graphene was chosen as a basis for one type of substrate due to its unique physical properties [30,31]. The interest in graphene is mainly due to its biocompatibility and ability to quench photoluminescence [32,33].

As previously shown by near-field and two-photon luminescence microscopy [22,25], the strongest FE is generated near the percolation threshold. The presence of such strong FE has recently allowed the realization of the color printing and laser-induced modification of local resonance properties in near-percolation metal films [34,35,36]. However, there are still no systematic studies of SERS signal dependence on the thickness of thin films near and far from the percolation threshold and applicability of such thin films for SERS. In papers where thin films of gold and silver were used for SERS application [37,38,39], the percolation threshold wasn’t clearly defined and/or the obtained results were not tied to such a concept as the percolation threshold. This is important because the percolation threshold can vary and depends on factors such as the substrate, deposited metal, methods of deposition, the deposition rate. Thus, the percolation threshold can vary and can be uniquely identified by electrical and/or optical methods [22,23,24,40,41,42].

In this research, we focus on a comprehensive analysis of ultrathin gold films with thicknesses close to the percolation threshold, deposited on SiO_2_/Si wafers with and without a single-layer CVD graphene for SERS applications.

## 2. Materials and Methods

### 2.1. Sample Fabrication

The ultrathin gold films with thicknesses ranging from 3 to 10 nm with a step size of 1 nm were deposited onto two types of substrates. The first type was a silicon wafer with a thin 90 nm thick layer of SiO_2_ on the surface (SiO_2_/Si). The second one was the same silicon wafer, but with a single-layer of CVD graphene, which covered more than 95% of the substrate area (graphene/SiO_2_/Si). Before metal deposition, the graphene substrates were annealed at 250 °C in a vacuum chamber 10^−6^ Torr for 1 h to remove residual PMMA and water. The thin gold films for all types of substrate were deposited in one mode by electron beam evaporation in a Nano Master NEE-4000 (NANO-MASTER Inc., Austin, TX, USA) installation at a high vacuum, with the pressure of residual gases in the chamber being no more than 5 × 10^−6^ Torr and a deposition rate of 0.5 Å/s, at room temperature (23 °C). Each thickness of the gold film was deposited simultaneously for both types of substrate in one cycle. As a material for deposition, granules of gold produced by Kurt J. Lesker with a purity of 99.999% were used. The quoted “thickness” of the gold films are the average nominal coverage values measured by the quartz oscillator.

### 2.2. Scanning Electron, Atomic Force Microscopy and Electrical Measurements

The fabricated films were subsequently imaged by scanning electron microscope (SEM) JEOL JSM-7001F (JEOL Ltd., Tokyo, Japan). The thickness of the films and their roughness were measured by an atomic force (AFM) neaSNOM Microscope (neaspec GmbH, Munich, Germany). Electrical measurements were carried out using a 4-probe station (Jandel Engineering Ltd., Linslade, UK) from Jandel with a collinear geometry of the location of the probes and started with 10 nA, gradually increasing the current to 100 μA.

### 2.3. Ellipsometry Characterization

The dielectric function spectra of the Au thin film were evaluated from data measured using a variable-angle spectroscopic ellipsometer (WVASE^®^, J. A. Woollam Co., Lincoln, NE, USA) operating in the wavelength range of 280–3300 nm. The data were collected at multiple angles of incidence from 65° to 75° with a step of 5° and the optical constants of the gold films were obtained by point-by-point fitting of the ellipsometry spectra with 10 nm steps [43]. To be included in the ellipsometry model, the optical constants for graphene were taken from the work [44].

### 2.4. Raman Characterization

The experimental setup used for Raman measurements was a confocal scanning Raman microscope Horiba LabRAM HR Evolution (HORIBA Ltd., Kyoto, Japan). All measurements were carried out using linearly polarized excitation at a wavelength of 632.8 nm, 300 lines/mm diffraction grating, and ×100 objective (N.A. = 0.90), whereas we used unpolarized detection to have a significant signal-to-noise ratio. The spot size was ~0.43 μm. The Raman spectra were recorded with 0.26 mW incident powers and an integration time of 1 s at each point. The statistics were collected from 15 × 15 points maps with a step of 0.8 μm from at least 10 different places of the sample. These scan parameters were selected as a compromise between minimum damage/bleaching of the dye molecules and significant signal-to-noise ratios. Raman dye Crystal Violet (CV) was used for the Raman characterization. Directly before the Raman measurement, the gold films deposited on two substrates (SiO_2_/Si and graphene/SiO_2_/Si) were covered by an aqueous 10^−6^ mol/L solution of CV for approximately 1 h and subsequently gently blown dry with compressed air.

## 3. Results

**Figure 1:**
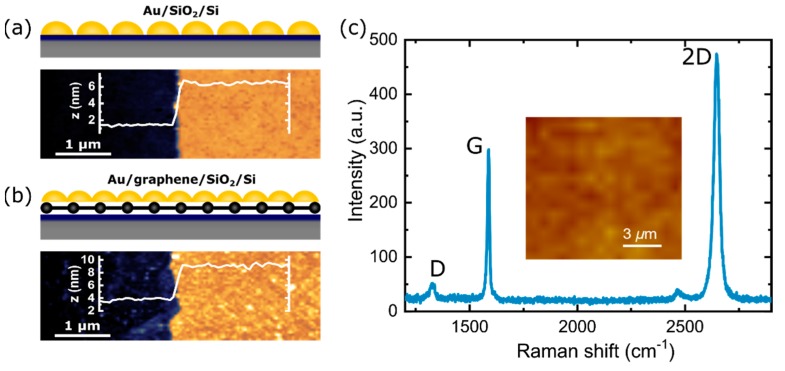
Schematic (top) and atomic force microscopy (AFM) (bottom) images and their cross-section of the edge of the gold film with a thickness of 4 nm deposited on (**a**) SiO_2_/Si and (**b**) graphene/ SiO_2_/Si substrates. (**c**) Raman spectra of monolayer CVD graphene. The insert in (**c**) is a Raman map of graphene before the deposition of the gold film.

The ultrathin gold films with thicknesses ranging from 3 to 10 nm with a step of 1 nm were deposited onto two types of substrate—SiO_2_/Si (Figure 1a) and graphene/ SiO_2_/Si (Figure 1b). The quality of monolayer graphene [45,46] was assessed with Raman spectroscopy (Figure 1c). From the optical image of the substrate surface and image obtained by Raman microscope, it can be seen that graphene uniformly covers the surface of the substrate without cracks and voids (Figure S1).

**Figure 2:**
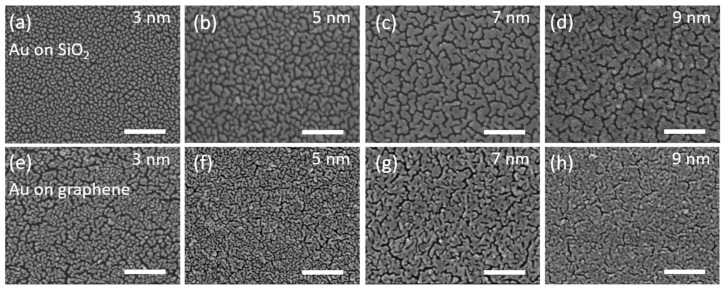
SEM images of the morphology the gold films deposited on the SiO_2_/Si substrate with thicknesses (**a**) 3 nm (**b**) 5 nm (**c**) 7 nm (**d**) 9 nm and on the graphene/SiO_2_/Si substrate with thicknesses (**e**) 3 nm (**f**) 5 nm (**g**) 7 nm (**h**) 9 nm. The scale bar is 200 nm.

The ratio of G and 2D bands (~0.6) indicates the single layer of graphene [46,47], and the ratio of the D and G bands (~0.09) indicates a low concentration of defects [47,48]. Additionally, the 2D band exhibits a single Lorentzian peak, while in the case two or more layers, it has broadband, which can be fitted by several Lorentzian peaks. Therefore, the profile of the 2D band is also used to identify monolayers [46]. All thicknesses of the deposited gold films were monitored both by a quartz sensor in the deposition installation and by independent measurements of the thickness by AFM at the border with a scratch (Figure 1a,b). SEM images of gold films with thicknesses of 3, 5, 7 and 9 nm (Figure 2) deposited on graphene/ SiO_2_/Si and SiO_2_/Si substrates show thin-film growing dynamics. The gold clusters grow faster along the substrate plane than in height. So, the individual clusters gradually begin to form a labyrinthine structure with increasing thickness and finally constitute an almost continuous film on both substrates. This can be seen in more detail in Figure S2, where SEM images for the films with thicknesses from 3 to 10 nm and a step of 1 nm are presented. Metal–graphene contact has been studied in some detail [49], and therefore it is known that the growth of metals on the surface of graphene differs markedly from the growth of metals on SiO_2_/Si substrates. Figure 2 and Figure S2 reflect this difference in film formation. Gold films with the same thickness deposited on graphene, consist of clusters with smaller sizes compared to gold films deposited on pure SiO_2_/Si substrates (Figure 2a–d). The distances between these clusters are also less than for clusters formed on substrates without graphene. Thus, a gold film on graphene covers a larger area of the substrate compared to a film on a SiO_2_/Si substrate of the same thickness. This could be due to the greater adhesion of gold and SiO_2_ in comparison to the adhesion between gold and graphene [49].

Routinely, the thickness of the deposited films is estimated by the quartz sensor in an electron beam evaporation installation, but the real thickness can be different. For the gold films, the quoted “thickness” is the average nominal coverage measured by the quartz oscillator. We accurately measured the thickness of the gold films and their roughness by AFM and compared how the obtained values correlate with the detector readings. The results of the AFM measurements are presented in Table 1. As can be seen from the table, the sensor values and the measurements by AFM can vary significantly. This is especially noticeable for thicknesses up to the percolation threshold. In cases when the thickness increases and a continuous film is practically formed, the thicknesses measured by AFM are closer to the sensor data/results. Apparently, this behavior can be explained by the density of “packing” for the metal on the surface. The packing density for thicknesses of metal up to the percolation threshold can be highly dependent on the adhesion between the metal and the substrate. In this case, the metal continues to build up thickness on the clusters, while slowly filling the voids between them. Whenever a continuous film is mostly formed, the metal is already deposited onto the formed film, and this can occur more evenly. If the present tendency for the thicknesses (measured using an AFM and with a quartz sensor) before and after the percolation threshold looks common for both types of substrates, then in the case of roughness the other behavior is observed. The example of typical AFM images and their profiles presented for a thickness of 4 nm (Figure 1a,b), demonstrate that the roughness of films on graphene/SiO_2_/Si substrate is greater than on SiO_2_/Si. The data in Table 1 show the distinction in the roughness of films on different substrates.

**Figure 3:**
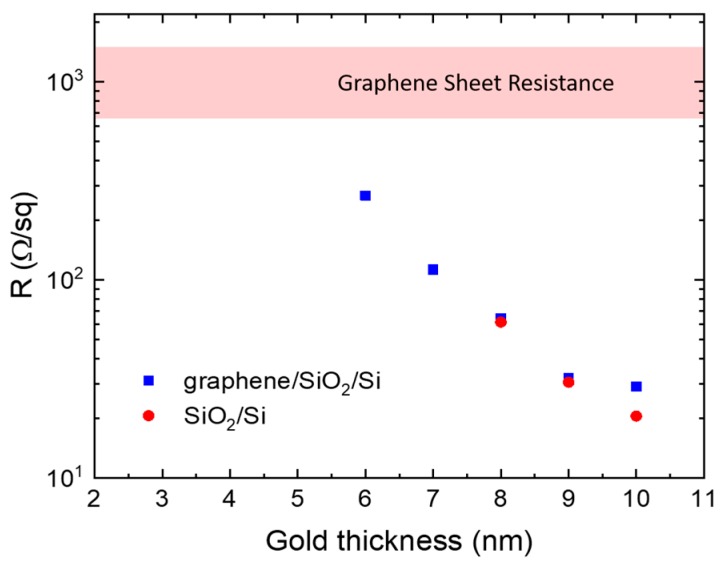
Dependence of the sheet resistance of gold films on their thickness for the two types of substrates.

Although we have shown the difference in thicknesses between those measured by AFM and by the quartz sensor, in the further representation of the experimental data we used the thicknesses given by the quartz sensor. Before the Raman measurements, we found a percolation threshold for deposited films by electrical and optical methods. The percolation threshold for films on the SiO_2_/Si substrate can be determined by sheet resistance measurements [23,41]. The experimental setup can be seen in the Supplementary Materials (Figure S3). The average sheet resistance for each of the thicknesses of the gold films is shown in Figure 3. The gold films deposited on a SiO_2_/Si substrate begin to conduct at thicknesses of 8 nm. Therefore, we can assume that the percolation threshold for such films is between 7 and 8 nm. Because graphene has very high electrical conductivity, the definition of the percolation threshold for the graphene/SiO_2_/Si substrate is more complicated than for previous samples, since this type of sample has conductivity at all thicknesses due to graphene. The sheet resistance of graphene was measured and the value ranged from 700 to 1600 Ω/sq. For films on graphene/SiO_2_/Si substrate, conductivity is observed over the entire range, but films can be considered percolated when their sheet resistance is much lower than the sheet resistance of graphene. According to this criterion, we can estimate that the percolation threshold is between 5 and 6 nm. Mostly, electrical measurements are enough to uniquely determine the film thickness at which there is a percolation threshold.

However, as we cannot uniquely determine these thicknesses for the gold films on the graphene/SiO_2_/Si substrate, we used ellipsometry as an additional method to determine the percolation threshold. Ellipsometry measurements give effective optical constants of gold films and can be used to determine the percolation threshold.

**Figure 4:**
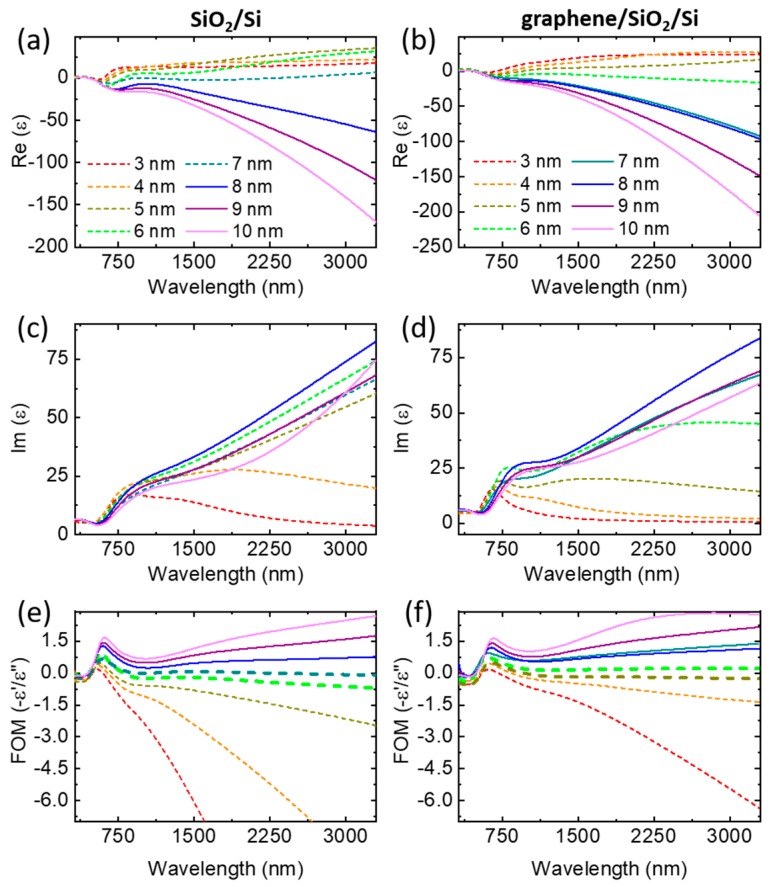
Dependence of the real and imaginary parts of the dielectric function on the thicknesses of gold films deposited on (**a**,**c**) SiO_2_/Si and (**b**,**d**) graphene/SiO_2_/Si substrates. FOM for gold films on (**e**) SiO_2_/Si and (**f**) graphene/SiO_2_/Si substrates. The dashed and solid lines correspond to percolated and continuous films, respectively.

The dielectric function spectra of the gold thin films [50,51] were evaluated from data (for an exemplified spectrum see Supplementary Materials Figure S4) measured using a variable-angle spectroscopic ellipsometer over a wavelength range from 280 to 3300 nm and are presented in Figure 4. The real part of the dielectric function of gold becomes less than zero in all measured wavelength range, for the films on SiO_2_/Si substrate starting from 8 nm (Figure 4a) and starting from 6 nm for the films on graphene/SiO_2_/Si substrate (Figure 4b). This means that the behavior of these curves (plotted as solid lines) is getting closer to the values of a continuous metal film. Analyzing the behavior of the curves which characterize the imaginary part of the dielectric function, it is clear to see that with an increase in the thickness of the gold films the curves take a different form all the time and transients occur (dashed lines), but starting from a certain thickness the curves take the same form, and the transients stop (solid lines). For the films on SiO_2_/Si substrate, the curves exhibit more typical behavior for the continuous film starting from 7 nm (Figure 4c) and for films on graphene/SiO_2_/Si substrate after 6 nm (Figure 4d).

In order to compare the metallic optical properties of films on two substrates, we calculated the plasmonic figure of merit (FOM), which is equal to −ε’/ε” and is presented in Figure 4e,f. FOM curves on the graph become higher as the film thicknesses increase and their specific behavior clearly demonstrates a difference in the quality [51] of the films on SiO_2_/Si (Figure 4e) and graphene/SiO_2_/Si (Figure 4f) in the infrared range for thicknesses from 3 to 7 nm. Starting from 8 nm for SiO_2_/Si and from 7 nm for graphene/SiO_2_/Si, the slope of curves in the infrared region changes sign from negative to positive. From this, we concluded that the percolation threshold for films on the SiO_2_/Si substrate is between 7 and 8 nm, which correlates well with the electrical measurements.

**Figure 5:**
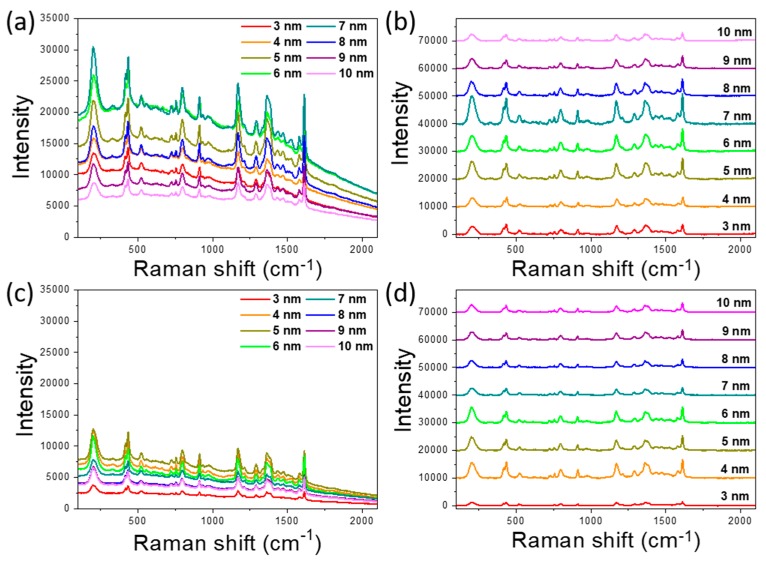
SERS spectra of Crystal Violet (CV) absorbed on the gold films with thicknesses from 3 to 10 nm, deposited on (**a**,**b**) SiO_2_/Si and (**c**,**d**) graphene/SiO_2_/Si substrates. (**b**,**d**) the SERS spectra of CV after subtracting the baseline.

Since the data obtained by ellipsometry have a good agreement with the electrical measurements for the films on the SiO_2_/Si, we can apply similar reasoning to estimate the percolation threshold for the films on the graphene/SiO_2_/Si substrate. The estimation exhibits the value between 6 and 7 nm, which is close to the region estimated by the electrical measurements. Thus, based on the measurements mentioned above, we can conclude that the percolation threshold for our gold films on the SiO_2_/Si substrate is between 7 and 8 nm and for the gold films on the graphene/SiO_2_/Si substrate is between 6 and 7 nm. In order to compare the hybrid graphene/gold substrates and substrates without graphene and estimate the enhancement factor of fabricated films, we used Raman microscopy. After the abovementioned measurements, all of the samples were covered by Raman dye CV (see Section 2). This Raman-active dye is chosen for its well-known and well-characterized properties. CV is a resonant dye for our excitation wavelength and can be a good marker to estimate the luminescence quenching. The dependence intensity of the SERS spectra on the gold film thickness is seen in Figure 5. The intensity gradually increases with increasing gold thickness from 3 nm to 7 nm in the case of films on the SiO_2_/Si (Figure 5a,b) and from 3 to 6 nm for the films on the graphene/SiO_2_/Si substrates (Figure 5c,d), respectively. The increased intensity of the SERS spectra for these films corresponds to increased FE, due to a decreased gap between the clusters. The maximum SERS signal was obtained for the gold films with thicknesses of 7 nm on the SiO_2_/Si and of 6 nm on the graphene/SiO_2_/Si substrates. This corresponds to the thicknesses defined as the percolation threshold by electrical and optical measurements. This is the case when the interparticle spaces lead to the formation of “hot spots” in the nano-gaps between clusters and as a result, to maximizing the intensity of the SERS spectra. Then, the intensity of the signal begins to decrease with increasing film thickness and reaches the minimum at a thickness of 10 nm for both types of substrate.

It can be expected that the intensity of the SERS signal should also be high for the thicknesses of adjacent films closest to the percolation threshold. These expectations are met for some thicknesses below to the percolation threshold; however, when the film thickness is above, the signal intensity drops down (Figure 5b,d). This can be explained due to the fact that up to the percolation threshold, the signal intensity of SERS depends on the number of “hot spots”, the size and shape of clusters, and the distance between them. In the case when the film turns into a continuous film, the number of hot spots drops sharply, as the part of clusters merge and form a continuous film. However, the SERS signal is still present and contribution to which gives the roughness, as well as the still presence of not merged individual clusters.

Additionally, we would like to draw attention to one interesting and important feature, the strong correlation between the calculated plasmonic FOM and measured SERS spectra. Indeed, the maximum SERS signal for both types of substrate (Figure S5) corresponds to zero slopes of the FOM curves in the infrared region, which are highlighted in bold (Figure 4e,f). For the island films, the slope of the curve in the infrared region takes a negative value and monotonically increases with increasing thickness, and for already percolated films, the slope takes a positive value. So, we suppose that the FOM curves can be used as a criterion for finding the percolation threshold and, accordingly, the film thicknesses that contribute to the maximum enhancement factor (EF).

The comparison of the obtained Raman spectra clearly demonstrates the difference in the level of fluorescence for the hybrid graphene/gold substrates and substrates without the graphene layer. In the presence of graphene, the luminescence level is much lower than without it (Figure 5a,c). The estimation shows that the presence of graphene on the substrate contributes to quench luminescence of CV by an average of ~60%. Note that for the gold thicknesses of 8–10 nm, the level of luminescence is almost the same for both SiO_2_/Si and graphene/SiO_2_/Si substrates. For these thicknesses, films are already continuous, and there is no interaction between the dye molecules and graphene.

For the calculation of enhancement, we use the analytical enhancement factor (EF) expression, which quantifies how much more signal can be expected from SERS in comparison with normal Raman for the same experimental parameters [52]. The average EF is determined by comparing the signals recorded from CV at a concentration of 10^−2^ mol/L on a silicon substrate, with the signals obtained with a concentration 10^−6^ mol/L of CV on the gold films with thicknesses from 3 to 10 nm deposited on different substrates. The following relation was used:

(1) $$EF\;=\;\frac{I_{SERS}}{I_{ref}}\frac{C_{ref}}{C_{SERS}},$$

where $I_{SERS}$ and $I_{ref}$ represent background-subtracted intensities of the 1626 cm^−1^ band for CV adsorbed on the gold films and the SiO_2_/Si substrate. $C_{SERS}$ and $C_{ref}$ represent the corresponding concentrations of CV on this substrate. The maximum EF was obtained for the film thicknesses, which were determined as thicknesses near the percolation thresholds. The EF of these thin gold films are estimated to be ~1.1 × 10^5^ (for the 7 nm gold film deposited on the SiO_2_/Si substrate) and ~6.0 × 10^4^ (for the 6 nm gold film deposited on the graphene/SiO_2_/Si substrate). Thus, the comparison of the hybrid graphene/gold substrates and substrates without graphene shows that, although the level of the SERS signal for the substrate with graphene is almost the same or even slightly lower, the ability to quench of luminescence provides a great advantage over other substrates. There is another interesting feature. If we compare the EF for the film with thickness 7 nm (this thickness is the percolation thresholds for the case of gold on SiO_2_/Si substrate) on SiO_2_/Si (EF ~ 1.1 × 10^5^), and graphene/SiO_2_/Si substrates (EF ~ 2.1 × 10^4^), a significant difference can be observed. Indeed, the presence of only one graphene layer on a SiO_2_/Si substrate can affect the thickness of the percolation threshold for the gold films and leads to the difference in SERS signal in near one order of magnitude.

## 4. Conclusions

In summary, this article presents a detailed study of semi-continuous gold films near the percolation threshold deposited on two types of substrate—SiO_2_/Si and graphene/SiO_2_/Si. We demonstrated that the thickness of the deposited gold films measured by AFM and sensor values can vary significantly. The percolation threshold has been determined by two independent experimental methods as a four-probe measurement of the sheet resistance and spectroscopic ellipsometry. These methods show good agreement with each other for both types of substrate with and without graphene. According to them, the thicknesses of the percolation threshold is between 7 and 8 nm for the films on the SiO_2_/Si substrate and between 6 and 7 nm on the graphene/SiO_2_/Si substrate. The maximum SERS signal was obtained for the gold films with thicknesses near the percolation threshold in the case of both types of substrate and amounted to be ~6.0 × 10^4^ (graphene/SiO_2_/Si) and ~1.1 × 10^5^ (SiO_2_/Si), respectively. SERS measurements of the dye Crystal Violet demonstrated the ability of graphene to effectively quench luminescence by an average of ~60%. Thus, the substrates with and without graphene show comparable SERS signals. However, the presence of graphene shows the ability to quench luminescence, which provides a great advantage over other types of SERS substrates. Our results demonstrate that the high intensity of the SERS signal is also observed for the films thicknesses adjacent with the percolation threshold, but only for thicknesses slightly below. Additionally, we show a strong correlation between FOM and intensity of SERS spectra. The maximum EF for both types of substrate corresponds to zero slopes of the FOM curves in the infrared region. We propose to use FOM curves as an additional criterion for finding the percolation threshold and the thicknesses of the films that exhibit the maximum EF. We believe that the reported results will be interesting for SERS applications, especially for bio- and molecular sensing.

## Figures and Tables

**Table 1:** Measured thicknesses of gold films using AFM in comparison with quartz sensor readings in an electron beam evaporation installation.

h (Quartz Sensor), nm	Graphene/SiO_2_/Si		SiO_2_/Si	
	h, nm	MSE, nm	h, nm	MSE, nm
3	4.5	0.8	3.4	0.4
4	5.2	0.7	5.1	0.5
5	6.9	0.7	5.9	0.4
6	7.9	0.8	6.7	0.4
7	8.1	0.7	7.5	0.3
8	8.8	0.7	8.3	0.4
9	10.1	0.6	9.5	0.4
10	10.9	0.7	10.3	0.4

## Supplementary Materials

The following are available online at https://www.mdpi.com/2079-4991/10/1/164/s1, Figure S1: Image of the substrate with CVD graphene in white light; Figure S2: SEM images of gold films with thicknesses from 3 to 10 nm deposited on (a)–(h) SiO_2_/Si and (i)–(p) graphene/SiO_2_/Si substrates respectively; Table S1: Table with averaged parameters of gold films deposited on graphene/SiO_2_/Si substrate; Table S2: Table with averaged parameters of gold films deposited on SiO_2_/Si substrate Figure S3: The schematic representation of the experimental setup used for the resistance measurements; Figure S4: An example of measured (dashed lines) and calculated (solid lines) from the fit ellipsometry parameters Ψ and Δ, used for Figure 4: Figure S5: The dependence of EF on the thicknesses of gold film (plotted with an integrated intensity of 1626 cm^−1^ Raman mode).
