# Supplementary material for: Surface-Enhanced Raman Spectroscopy on Hybrid Graphene/Gold Substrates near the Percolation Threshold
Source: Nanomaterials (Basel). 2020 Jan 17;10(1):164. doi: 10.3390/nano10010164 (PMC7022774; doi:10.3390/nano10010164)
Supplement: Supplementary file 1 [file nanomaterials-10-00164-s001.pdf]

nanomaterials

MDPI

Supplementary Materials

Surface-Enhanced Raman Spectroscopy on Hybrid Graphene/Gold Substrates near the Percolation Threshold

Dmitry E. Tatarkin [1,*], Dmitry I. Yakubovsky [1], Georgy A. Ermolaev [1,2], Yury V. Stebunov [1], Artem A. Voronov [1], Aleksey V. Arsenin [1], Valentyn S. Volkov [1] and Sergey M. Novikov [1,*]

[1] Center for Photonics and 2D Materials, Moscow Institute of Physics and Technology (MIPT), 141700 Dolgoprudny, Russia; dmitrii.yakubovskii@phystech.edu (D.I.Y.); ermolaev-georgy@yandex.ru or georgy.ermolaev@skoltech.ru (G.A.E.); stebunov@phystech.edu (Y.V.S.); voronov.artem@gmail.com (A.A.V.); arsenin.av@mipt.ru (A.V.A.); vsv.mipt@gmail.com (V.S.V.)

[2] Skolkovo Institute of Science and Technology, 121205 Moscow, Russia

* Correspondence: tatarkin.de@phystech.edu (D.E.T.); novikov.s@mipt.ru (S.M.N.); Tel.: +7-999-150-1442 (D.E.T.); +7-903-236-0487 (S.M.N.)

Received: 19 December 2019; Accepted: 14 January 2020; Published: date

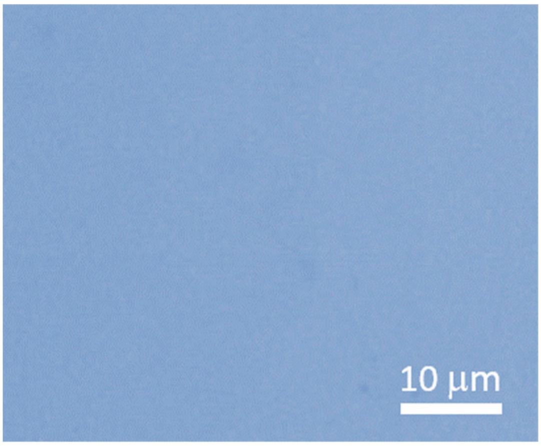

Figure 1. Image of the substrate with graphene in white light.

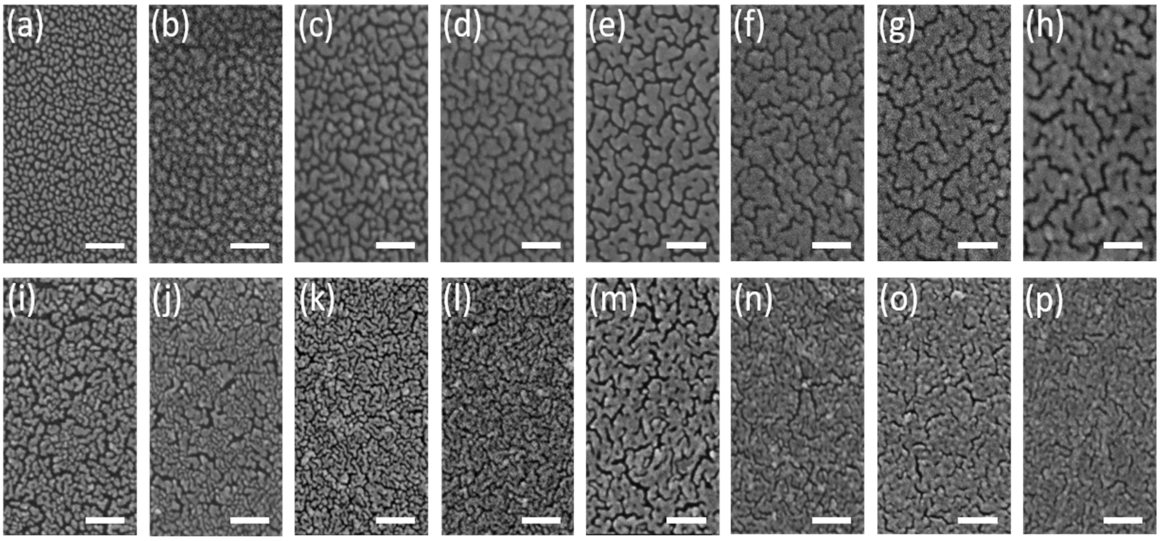

Figure S2. SEM images of gold films with thicknesses from 3 to 10 nm deposited on a)-h) SiO_2/Si and i)-p) graphene/SiO_2/Si substrates, respectively. The scale bar is 100 nm.

Table S1. Table with averaged parameters of gold films deposited on graphene/SiO_2/Si substrate

thickness (nm)	occupancy (%)	the average size of NPs (nm)
3	53.9	11.1
4	58.8	12.8
5	67.1	14.8
6	71.3	15.3
7	75.1	16.1

Table S2. Table with averaged parameters of gold films deposited on SiO_2/Si substrate

thickness (nm)	occupancy (%)	the average size of NPs (nm)
3	50.2	11.3
4	56.8	16.2
5	60.5	24.0
6	65.8	-
7	69.5	-

Sheet resistance measurement system (Yandel RM3000) is based on four tungsten probes having distance between each of them 625 μkm and radius of needle 40 μkm. Probes were approached to a metal film providing ohmic contact, what was confirmed by measuring the resistance value $R_{sq.}$ at different currents in the range of 10-100 mA. Each fabricated film, which is conductive, demonstrated voltage-current dependence corresponding to ohmic contact. The experimental setup can be seen in Figure S3

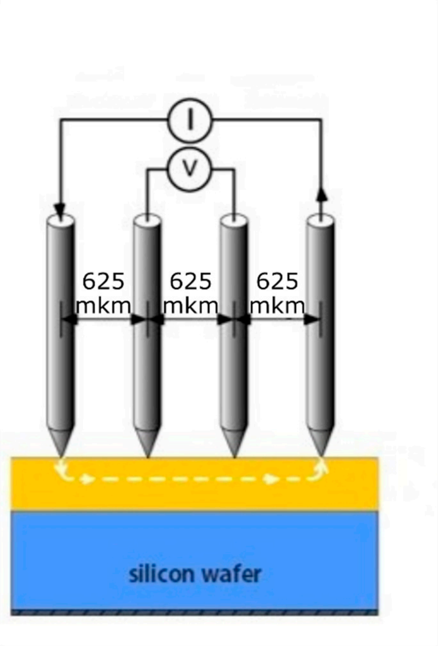

Figure S3. The schematic representation of the experimental setup used for the resistance measurements

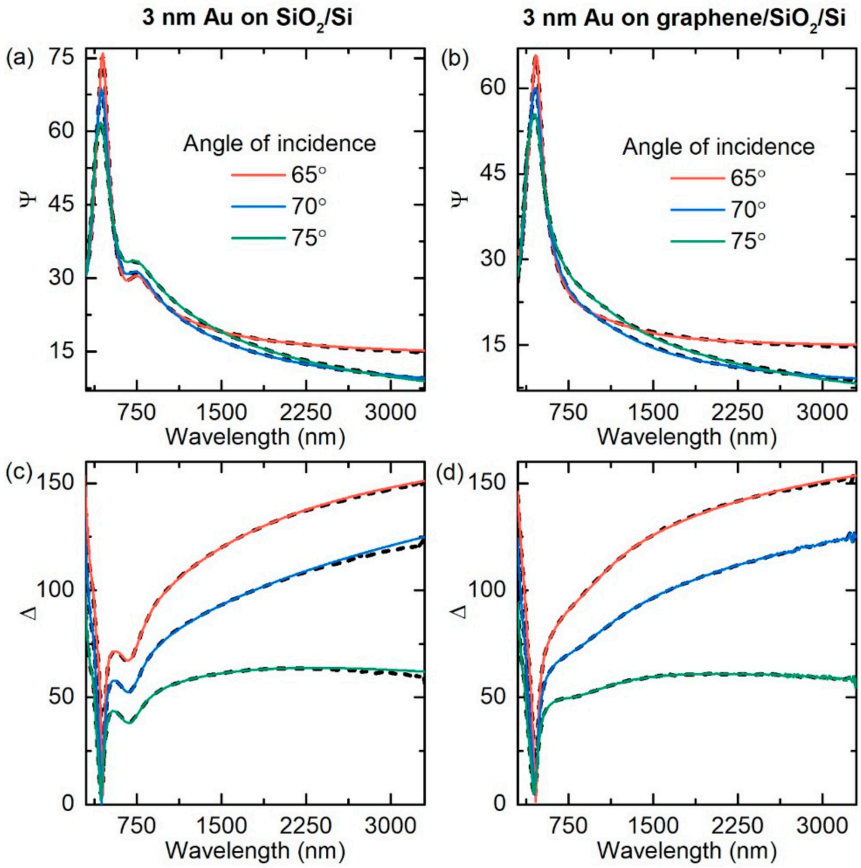

Figure S4. An example of measured (dashed lines) and calculated (solid lines) from the fit ellipsometry parameters ψ and Δ, used for Figure 4.

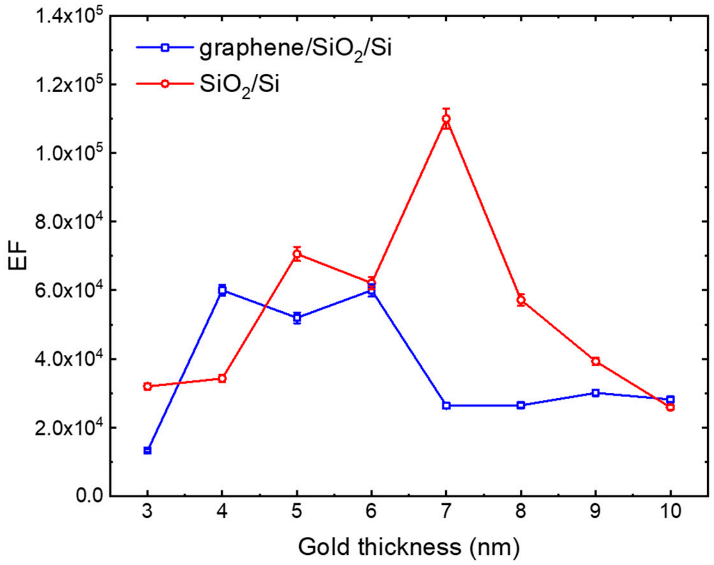

Figure S5. The dependence of EF on the thicknesses of the gold film (derived by the integrated intensity of 1626 cm^{-1} Raman mode)

© 2020 by the authors. Submitted for possible open access publication under the terms and conditions of the Creative Commons Attribution (CC BY) license (http://creativecommons.org/licenses/by/4.0/).
